# Hepcidin Response to Iron Therapy in Patients with Non-Dialysis Dependent CKD: An Analysis of the FIND-CKD Trial

**DOI:** 10.1371/journal.pone.0157063

**Published:** 2016-06-08

**Authors:** Carlo A. Gaillard, Andreas H. Bock, Fernando Carrera, Kai-Uwe Eckardt, David B. Van Wyck, Sukhvinder S. Bansal, Maureen Cronin, Yvonne Meier, Sylvain Larroque, Simon D. Roger, Iain C. Macdougall

**Affiliations:** 1 Department of Nephrology, University Medical Center Groningen, University of Groningen, Groningen, the Netherlands; 2 Department of Nephrology, Kantonsspital, Aarau, Switzerland; 3 Department of Dialysis, Eurodial, DaVita, Leiria, Portugal; 4 Department of Nephrology and Hypertension, University of Erlangen-Nürnberg, Erlangen, Germany; 5 Clinical Support Services, Davita Healthcare Partners, Denver, CO, United States of America; 6 Institute of Pharmaceutical Science, King’s College, London, United Kingdom; 7 Vifor Pharma Ltd, Glattbrugg, Switzerland; 8 Renal Research, Gosford, NSW, Australia; 9 Department of Renal Medicine, King’s College Hospital, London, United Kingdom; Medical University of Graz, AUSTRIA

## Abstract

Hepcidin is the key regulator of iron homeostasis but data are limited regarding its temporal response to iron therapy, and response to intravenous versus oral iron. In the 56-week, open-label, multicenter, prospective, randomized FIND-CKD study, 626 anemic patients with non-dialysis dependent chronic kidney disease (ND-CKD) and iron deficiency not receiving an erythropoiesis stimulating agent were randomized (1:1:2) to intravenous ferric carboxymaltose (FCM), targeting higher (400–600μg/L) or lower (100–200μg/L) ferritin, or to oral iron. Serum hepcidin levels were measured centrally in a subset of 61 patients. Mean (SD) baseline hepcidin level was 4.0(3.5), 7.3(6.4) and 6.5(5.6) ng/mL in the high ferritin FCM (n = 17), low ferritin FCM (n = 16) and oral iron group (n = 28). The mean (SD) endpoint value (i.e. the last post-baseline value) was 26.0(9.1),15.7(7.7) and 16.3(11.0) ng/mL, respectively. The increase in hepcidin from baseline was significantly smaller with low ferritin FCM or oral iron vs high ferritin FCM at all time points up to week 52. Significant correlations were found between absolute hepcidin and ferritin values (r = 0.65, p<0.001) and between final post-baseline increases in both parameters (r = 0.70, p<0.001). The increase in hepcidin levels over the 12-month study generally mirrored the cumulative iron dose in each group. Hepcidin and transferrin saturation (TSAT) absolute values showed no correlation, although there was an association between final post-baseline increases (r = 0.42, p<0.001). Absolute values (r = 0.36, p = 0.004) and final post-baseline increases of hepcidin and hemoglobin (p = 0.30, p = 0.030) correlated weakly. Baseline hepcidin levels were not predictive of a hematopoietic response to iron therapy. In conclusion, hepcidin levels rose in response to either intravenous or oral iron therapy, but the speed and extent of the rise was greatest with intravenous iron targeting a higher ferritin level. However neither the baseline level nor the change in hepcidin was able to predict response to therapy in this cohort.

## Introduction

Since its discovery in 2001 [1, 2], the peptide hormone hepcidin has been identified as the principal regulator of iron availability in the body. It maintains iron homeostasis by controlling intestinal absorption of dietary iron and release of iron from the liver and spleen [3]. Hepcidin acts by binding to, and inducing internalization of, the transmembrane iron transporter protein ferroportin, thus blocking export of iron from enterocytes, macrophages and parenchymal hepatocytes into the circulation. When iron availability is inadequate, low hepcidin levels promote enhanced intestinal iron uptake and release of stored iron. Hepcidin levels rise in response to iron repletion such that iron absorption and release are reduced. Stimulation of erythropoiesis, conversely, lowers hepcidin levels through increased production of erythroferrone by erythroblasts [4]. Furthermore, hepcidin levels are increased in the presence of chronic inflammation, such as in patients with chronic kidney disease (CKD) [5–8], contributing to the functional iron deficiency which is frequently observed in this patient population.

The key regulatory role of hepcidin has prompted interest in the possibility that it may represent a more reliable indicator of iron status than current biomarkers such as ferritin [3, 9, 10] and indeed may represent a useful therapeutic target [11]. As with ferritin, however, the predictive value of hepcidin is complicated by the influence of inflammation on hepcidin levels [3, 12], and by high intra-individual variation [13]. Data are conflicting regarding an association between hepcidin and the erythropoietic response to erythropoiesis stimulating agent (ESA) therapy [13–17], or iron therapy [13, 16, 18–22]. The longitudinal response of hepcidin levels to iron therapy has not been examined, nor are there any data comparing the short- or long-term hepcidin response to intravenous (IV) versus oral iron therapy. Additionally, the associations between hepcidin and other markers of iron status, or anemia, are poorly defined.

The Ferinject^®^ assessment in patients with Iron deficiency anemia and Non-Dialysis dependent Chronic Kidney Disease (FIND-CKD) study was a randomized, international, trial of IV ferric carboxymaltose (FCM) versus oral iron in patients with non-dialysis dependent CKD, anemia, and iron deficiency not receiving ESA therapy [23]. As part of the study protocol, hepcidin levels were measured in a subset of patients throughout the 12-month study. The current exploratory analysis was undertaken *post hoc* with the objective of determining the response of hepcidin levels to IV or oral iron treatment over time.

## Methods

### Study design

FIND-CKD was a 56-week, open-label, multicenter, prospective, randomized, three-arm study undertaken at 193 nephrology centers in 20 countries (ClinicalTrials.gov NCT00994318). The first patient visit took place in December 2009, with the final patient visit in January 2012. The study design has been described in full elsewhere [24] and is summarized here. The study protocol was approved at all participating sites (see S1 Appendix for a list of Ethics Committees). The study was conducted according to the principles of the Declaration of Helsinki and the ICH Guidelines for Good Clinical Practice. All patients provided written informed consent.

The primary endpoint of the study was time to initiation of other anemia management, specified as ESAs, blood transfusion, use of an alternative iron therapy, or occurrence of a Hb trigger (two consecutive Hb values <10 g/dL on or after week 8). Pre-planned analyses included the correlations between hepcidin and iron parameters (ferritin and transferrin saturation [TSAT]) at baseline and by visit, both for absolute values and for change from baseline. Other analyses reported here were performed *post hoc*.

### Study population

Full eligibility criteria have been published previously [24]. In brief, adult (≥18 years) patients with non-dialysis dependent CKD were enrolled if (a) at least one Hb level was 9–11 g/dL within four weeks of randomization, (b) any ferritin level was <100 μg/L, or <200 μg/L with TSAT <20%, within four weeks of randomization, (c) estimated glomerular filtration rate (eGFR) was ≤60 mL/min/1.73m^2^ (d) no ESA had been administered within four months of randomization, (e) no documented history of gastrointestinal intolerability to oral iron (e) no known active infection and baseline C-reactive protein (CRP) ≤ 20 mg/L.

### Study treatment

Patients were randomized in 1:1:2 ratio to high ferritin FCM: low ferritin FCM: oral iron. FCM dose (Ferinject^®^, Vifor International, St Gallen, Switzerland) in the high ferritin and low ferritin FCM groups was adjusted to target a ferritin level of 400–600 μg/L and 100–200 μg/L, respectively. An initial single dose was administered on day 0: 1000 mg iron as FCM in the high ferritin FCM group (500 mg iron on days 0 and 7 in patients weighing ≤66 kg) and 200 mg iron as FCM in the low ferritin FCM group if ferritin was <100 μg/L. During weeks 4 to 48, FCM was administered every four weeks in the high ferritin FCM group at a dose of 500 mg iron if ferritin was in the range 200 to <400 μg/L, and at a dose of 1000 mg iron if ferritin was <200 μg/L, and in the low ferritin FCM group at a dose of 200 mg iron if ferritin was <100 μg/L. In both groups, dosing was withheld if TSAT was ≥40%. Oral iron therapy consisted of commercially-available ferrous sulfate at a dose of 304 mg (100 mg of iron) twice daily.

Until week 8 post-randomization, patients were not to receive ESAs, blood transfusion or any anemia therapy other than study drug unless there was an absolute requirement.

### Hepcidin

All UK patients had hepcidin levels in serum samples measured at baseline (week 0) and at weeks 4, 8, 12, 24, 36 and 52 post-randomization. Hepcidin was measured at King’s College London in serum using ultra-high pressure liquid chromatography (UPLC) and a triple-quadruple mass spectrometer based on published methodology [25]. Due to a change of mass spectrometer, the assay has undergone a further validation [26]. The lower limit of quantitation was 0.1 ng/mL with less than 20% precision and accuracy.

### Statistical analysis

All patients who received at least one dose of randomized treatment (or according to the protocol were not treated due to ferritin level) and who attended at least one post-baseline visit were included in the intention-to-treat (ITT) population. Hepcidin data were analyzed for all ITT patients with a baseline hepcidin value and at least one post-baseline value (i.e. the 'hepcidin subpopulation'). The statistical analysis plan specified that hepcidin values were included only up to the point at which the primary endpoint was met (i.e. another anemia therapy was initiated) or the end of the study (for patients who did not meet primary endpoint).

All analyses were based on the natural scale (i.e. untransformed data). Summary statistics are provided for baseline and post-baseline hepcidin levels by treatment group. The change in hepcidin level from baseline to each post-baseline time point was compared within each treatment group using the Student paired t-test. The pairwise comparisons of treatment groups in the change in hepcidin values from baseline to all post-baseline visits were assessed by a mixed model with repeated measures procedure, using treatment group, baseline hepcidin value, visit, age at baseline and interaction between visit and treatment as covariates. The correlation between observed hepcidin levels versus ferritin, TSAT, Hb and estimated GFR (GFR) levels, and between changes in levels from baseline, was assessed by Pearson coefficients across patients in all treatment groups, using endpoint values (i.e. the last non-missing post-baseline value recorded). Correlations were analyzed similarly within each treatment group at weeks 8 and 24. Patient numbers were considered too low after week 24 for within-group analyses, although positive correlations in either FCM treatment arm prompted an additional assessment at week 52 or endpoint, and/or in pooled FCM groups. All assessments of correlations between hepcidin and ferritin, TSAT, Hb and eGFR included only values recorded on the same sample date. Ferritin and Hb values measurements were based on central analysis; TSAT values were obtained locally. eGFR based on locally-measured serum creatinine values was calculated based on the Modification of Diet in Renal Disease-4 (MDRD-4) equation.

As an exploratory analysis, the hazard ratios and associated 95% confidence intervals for the primary endpoint of the study (time to initiation of other anemia management or occurrence of hepcidin trigger [24]) were calculated using Cox proportional hazards modeling adjusting for (a) hepcidin at baseline (b) change in hepcidin to week 8 and (c) change in hepcidin to week 24.

All statistical analyses were performed using SAS Version 9.3 (SAS Institute Inc. SAS/STAT, Cary, NC, USA).

## Results

### Study population

**Fig 1:**
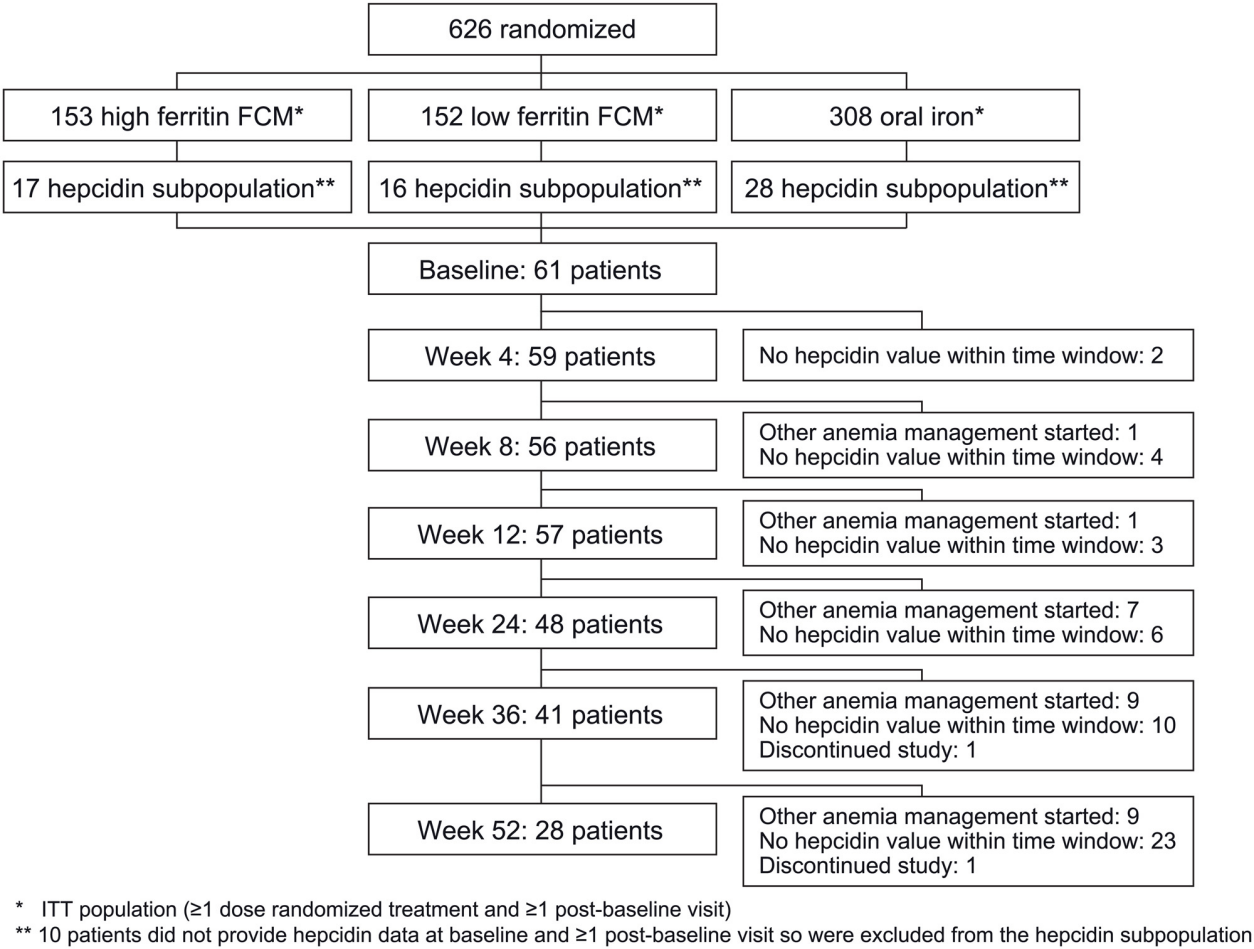
Patient disposition (hepcidin subpopulation). Disposition according to treatment group is shown in S1 Table.

In total, 613 patients formed the ITT population, of whom 71 patients were recruited in the UK and were candidates for the hepcidin substudy. Of these, 61 provided both a baseline and ≥1 post-baseline value and formed the hepcidin subpopulation (17 high ferritin FCM, 16 low ferritin FCM, 28 oral iron). During the 12-month study, a total of 10 patients received an alternative anemia therapy at some point (2 high ferritin FCM, 2 low ferritin FCM, 6 oral iron), and in these patients subsequent hepcidin values were excluded from analysis since this could have affected hepcidin concentrations. Overall, hepcidin data were available from 59, 56, 57, 48, 41 and 28 patients at weeks 4, 8, 12, 24, 36 and 52, respectively (Fig 1) (S1 Table).

The characteristics of the patients included in the hepcidin subpopulation (Table 1) showed no relevant differences to the rest of the study population [21].

**Table 1:** Patient demographics and baseline characteristics.

		Hepcidin subpopulation^a^ (n = 61)	Patients not included in hepcidin analysis (n = 552)
Age,years	Mean (SD)	66.1 (13.8)	69.4 (13.1)
Female gender	n (%)	35 (57.4)	346 (62.7)
White race	n (%)	46 (75.4)	538 (97.8)
Hb, g/dL	Mean (SD)	10.4 (0.8)	10.4 (10.7)
Ferritin, μg/L	Mean (SD)	55 (35)	57 (47)
TSAT, %^b^	Mean (SD)	18 (7)	16 (11)
CRP, mg/L^b^	Mean (SD)	6.7 (5.6)	5.7 (8.7)

^a^ Defined as all ITT patients with a baseline hepcidin value and at least one post-baseline value

^b^ Local laboratory

Hb, hemoglobin; CRP, C-reactive protein; SD, standard deviation; TSAT, transferrin saturation.

### Hepcidin levels over time

Mean (SD) hepcidin level at baseline was 4.0 (3.5), 7.3 (6.4) and 6.5 (5.6) ng/mL in the high ferritin FCM, low ferritin FCM and oral iron groups, respectively. These values were broadly similar to the normal range for healthy individuals of this age group based on the assay used in this analysis [25].

**Fig 2:**
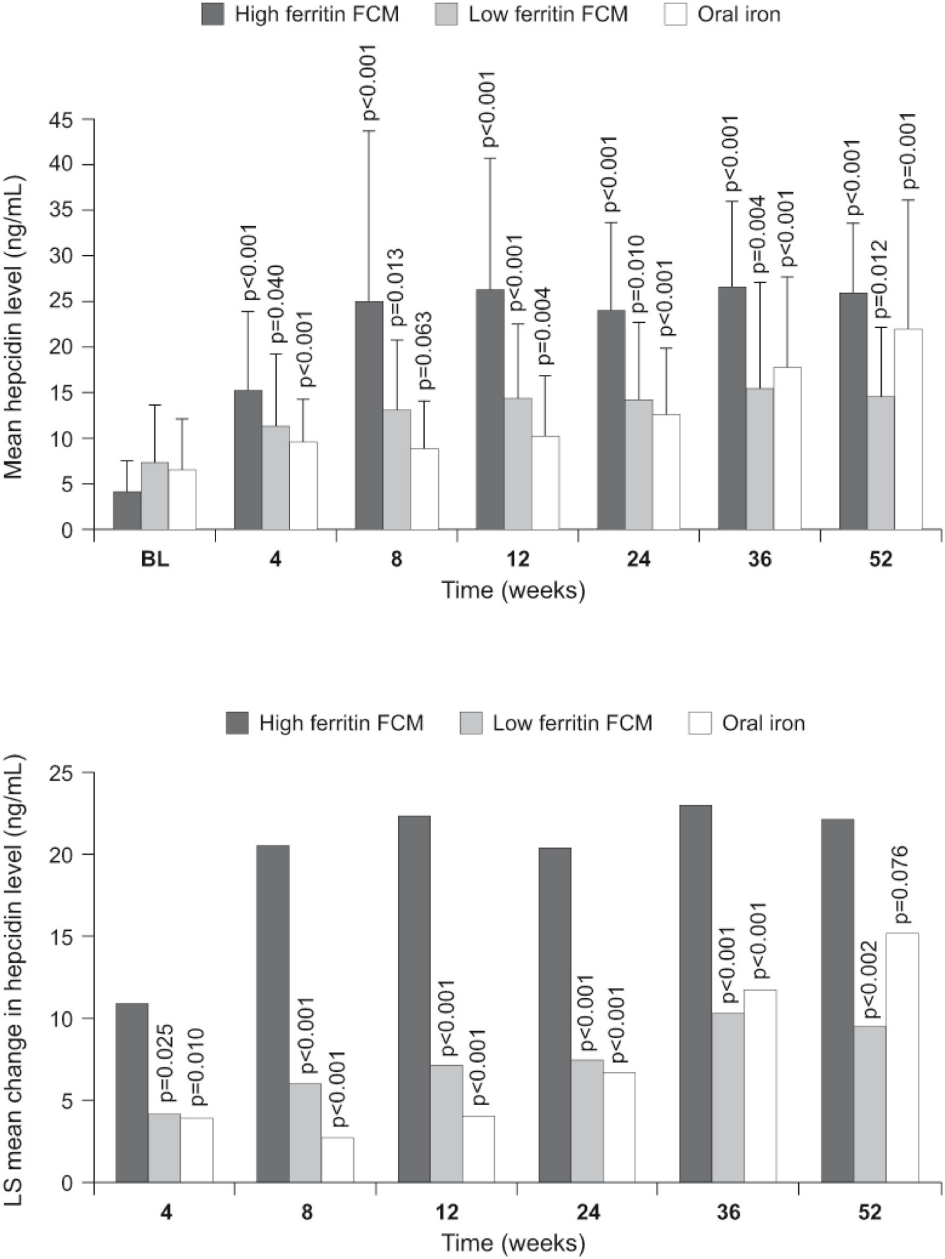
(a) Observed mean (SD) hepcidin level at each visit by treatment group. The p values indicate change from baseline (student paired t test). (b) Least square [LS] mean change in hepcidin level from baseline to each visit by treatment group by a mixed model with repeated measures procedure, with treatment group and baseline hepcidin as covariates. The p values refer to the comparison of change from baseline in the low ferritin FCM group or oral group versus the high ferritin FCM group. All differences between the low ferritin FCM and oral iron groups for the mean change in hepcidin level from baseline were non-significant (p>0.05). In both graphs, only values obtained prior to the start of alternative anemia therapy are included.

Post-baseline, the highest hepcidin values were observed in the high ferritin FCM group at all time points (Fig 2a). The change from baseline was statistically significant at all post-baseline visits in the high ferritin FCM group and the low ferritin group while for the oral iron group the change was significant except for week 8 (p = 0.063) (Fig 2a). The mean (SD) endpoint value (i.e. the last non-missing value recorded after baseline) was 26.0 (9.1), 15.7 (7.7) and 16.3 (11.0) ng/mL in the high ferritin FCM, low ferritin FCM and oral iron groups, respectively.

The increase in hepcidin was significantly smaller in the low ferritin FCM group and the oral iron group compared to the high ferritin FCM group at all post-baseline time points other than week 52 (Fig 2b). Other predictive factors for the increase in hepcidin were baseline hepcidin (p = 0.038) and time from start of treatment (i.e. study visit) (p<0.001). The post-baseline hepcidin increase was similar in the low ferritin FCM group and the oral iron group at all times (Fig 2b).

### Hepcidin level and iron dose

**Fig 3:**
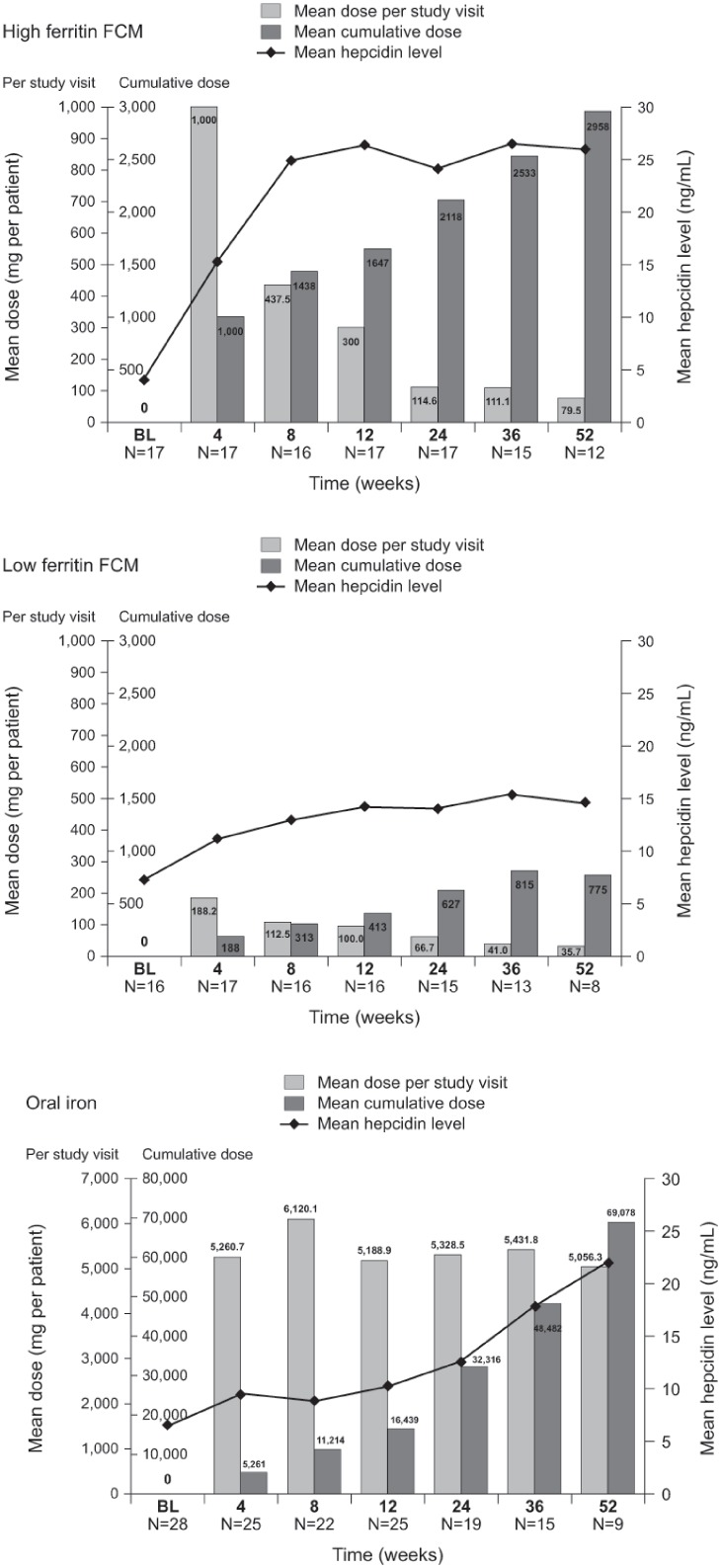
Mean hepcidin level and mean iron dose administered at each visit for (a) high ferritin FCM (b) low ferritin FCM (c) oral iron. Bars indicate iron dose per visit and cumulative iron dose, lines indicate mean hepcidin level. Only values obtained prior to the start of alternative anemia therapy are included BL, baseline; FCM, ferric carboxymaltose.

The change in hepcidin level over the 12-month study generally mirrored the cumulative iron dose administered in each of the three treatment groups (Fig 3). However, most FCM in the high ferritin group was administered at the early study visits (S1 Fig), as was a high proportion of FCM in the low ferritin group, whereas daily oral dosing was continued throughout the study (Fig 3). The mean hepcidin level increased rapidly and remained elevated after the first high ferritin FCM dose (Fig 3a), with a similar but less profound pattern of change in the low FCM group (Fig 3b). Hepcidin levels increased more slowly and progressively over the 52-week study in response to oral iron therapy (Fig 3c).

### Association between hepcidin level and markers for iron status

#### Serum ferritin

**Fig 4:**
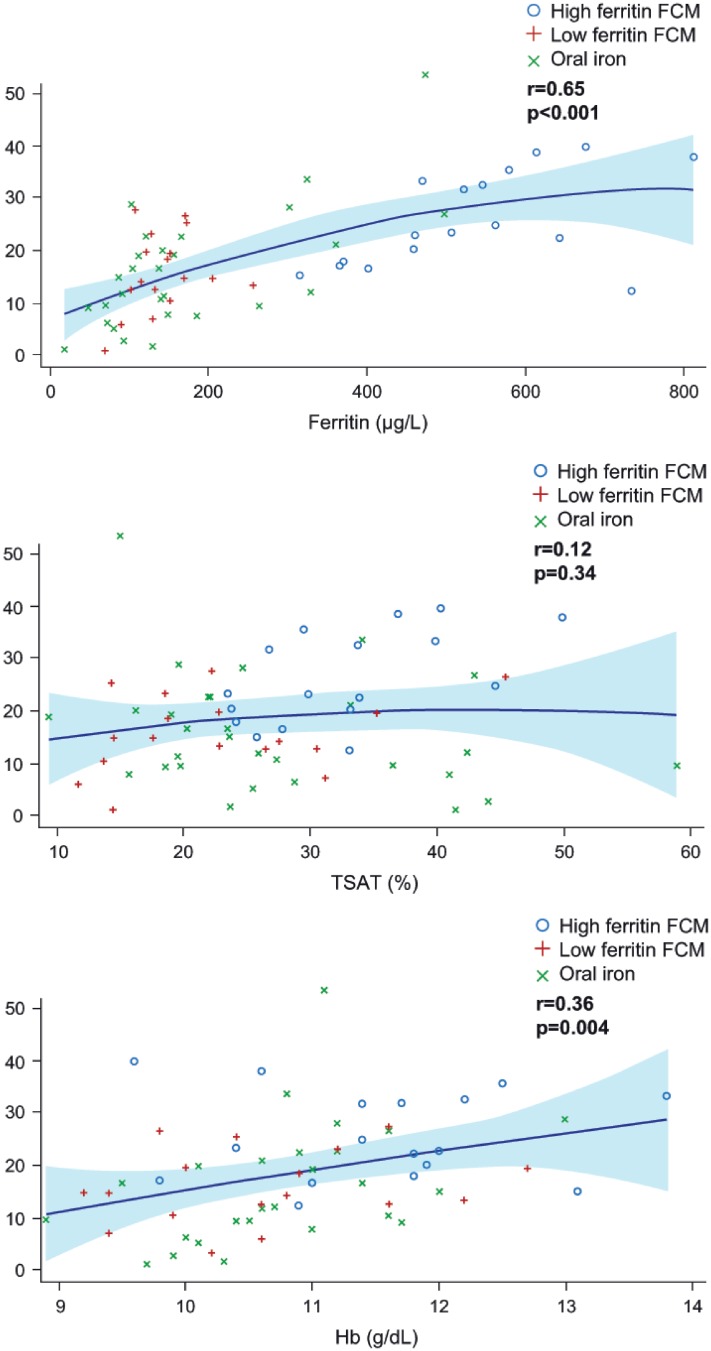
Correlation between hepcidin levels and (a) ferritin level (b) TSAT level (c) Hb level at study endpoint for the hepcidin substudy population. Only values with the same sample date, and obtained prior to the start of alternative anemia therapy, are included. Solid lines indicate the quadratic regression line. Shaded areas indicate 95% CI of the regression line.

**Fig 5:**
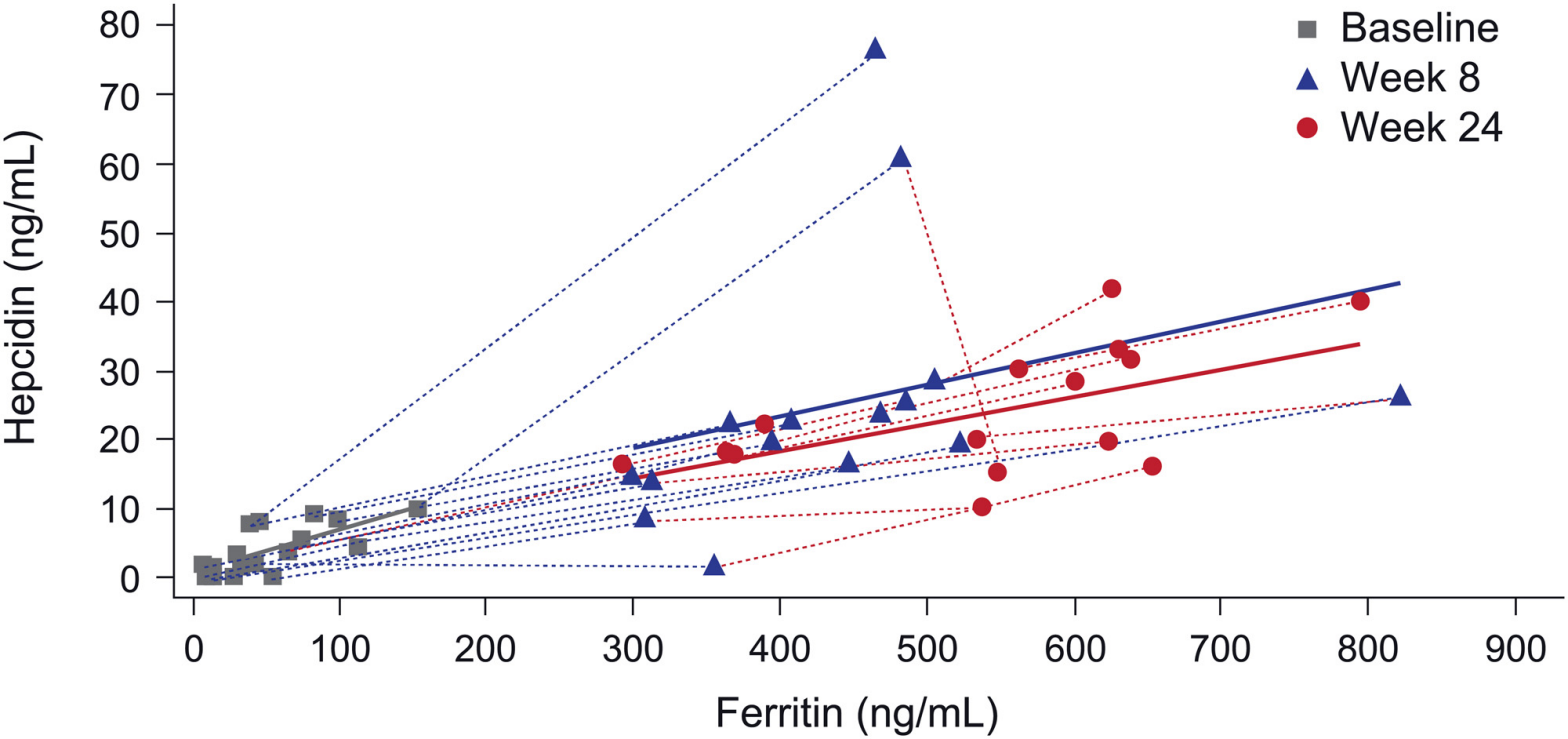
Correlation between hepcidin levels and ferritin level at baseline, week 8 and week 24 for patients in the high ferritin FCM group (n = 17). Only values with the same sample date, and obtained prior to the start of alternative anemia therapy, are included Correlation between hepcidin levels and (a) ferritin level (b) TSAT level (c) Hb level at study endpoint for the hepcidin substudy population. Only values with the same sample date, and obtained prior to the start of alternative anemia therapy, are included. Solid lines indicate the quadratic regression line. Shaded areas indicate 95% CI of the regression line. Correlation between hepcidin levels and (a) ferritin level (b) TSAT level (c) Hb level at study endpoint for the hepcidin substudy population. Only values with the same sample date, and obtained prior to the start of alternative anemia therapy, are included. Solid lines indicate the quadratic regression line. Shaded areas indicate 95% CI of the regression line.

In the total hepcidin substudy population, there was a significant correlation between hepcidin and ferritin levels at baseline (r = 0.43, p<0.001) and at the study endpoint (i.e. the last available post-baseline visit values) (r = 0.65, p<0.001) (Fig 4a). At higher ferritin levels the association between hepcidin and ferritin seems to plateau although the patient numbers at these high levels are too low to draw firm conclusions. There was also a close correlation between change in hepcidin and change in ferritin from baseline to the endpoint value (r = 0.70, p<0.001) (Table 2). Fig 5 shows the correlation between hepcidin and ferritin levels in the high ferritin FCM group at baseline, week 8 and week 24, illustrating that the correlation was similar at low or high ferritin levels (i.e. before or after the start of high ferritin FCM administration).

**Table 2:** Correlations between change in hepcidin levels from baseline versus change in serum ferritin, TSAT and Hb levels from baseline. Significant p values are shown in bold.

	Correlation with change in hepcidin from baseline		
	n	R	P value
**Change in serum ferritin from baseline, ng/mL**			
Endpoint values All patients	61	0.70	**<0.001**
Week 8			
High ferritin FCM	16	0.29	0.28
Low ferritin FCM	16	0.72	**0.001**
Oral iron	24	-0.34	0.11
Week 24			
High ferritin FCM	15	0.70	**0.004**
Low ferritin FCM	14	-0.04	0.88
Oral iron	19	0.23	0.33
**Change in TSAT from baseline, %**			
Endpoint values All patients	61	0.42	**<0.001**
Week 8			
High ferritin FCM	16	0.45	0.077
Low ferritin FCM	16	0.05	0.86
Oral iron	24	0.38	0.068
Week 24			
High ferritin FCM	15	0.67	**0.006**
Low ferritin FCM	14	0.30	0.30
Oral iron	18	0.03	0.92
**Change in Hb from baseline, g/dL**			
Endpoint values All patients	54	0.30	**0.030**
Week 8			
High ferritin FCM	9	0.09	0.81
Low ferritin FCM	15	-0.28	0.31
Oral iron	20	0.16	0.49
Week 24			
High ferritin FCM	13	0.53	0.064
Low ferritin FCM	13	-0.11	0.71
Oral iron	15	-0.27	0.33

Correlation estimates and p values were from the Pearson statistics (r)

Endpoint values represent the last non-missing post-baseline value recorded

All assessments included only values recorded on the same sample date. Only data prior to start of alternative anemia therapy are included.

FCM, ferric carboxymaltose; TSAT, transferrin saturation.

#### TSAT

Hepcidin did not correlate with TSAT at baseline in the overall hepcidin study population (r = -0.01, p = 0.91). Neither was any significant correlation observed for absolute hepcidin and TSAT based on endpoint values (r = 0.12, p = 0.34) (Fig 4b). The change in hepcidin versus the change in TSAT from baseline to the endpoint value, however, was significant (r = 0.42, p<0.001) but the correlation was less strong than for serum ferritin (Table 2). The correlations between hepcidin and TSAT remained significant based on endpoint values for the pooled FCM cohort, both for absolute values (r = 0.59, p<0.001; n = 33) and change from baseline (r = 0.69, p<0.001; n = 33).

#### Association between hepcidin level and Hb

At baseline, there was no association between absolute values for hepcidin and Hb values across all patients (r = 0.02, p = 0.88). There was a relatively weak, albeit significant, positive correlation for endpoint values (r = 0.36, p = 0.004) (Fig 4c) and between the change in hepcidin and change in Hb from baseline to the endpoint value (r = 0.30, p = 0.030) (Table 2).

#### Hepcidin as a predictor of response to iron therapy

Baseline hepcidin values and the mean hepcidin increase from baseline to week 8 and week 24 were calculated within each treatment group for the subsets of patients with or without an increase in Hb of >1g/dL, >0.5g/dL or 0 g/dL at the same time point. No association was observed between baseline hepcidin or hepcidin increase and the Hb increase in either FCM treatment group using any of the cut-off points. In the oral iron group, mean baseline hepcidin was lower in patients who achieved an increase in Hb of >1 g/dL versus patients who did not at week 8 (mean 0.1 ng/mL [n = 1] versus 7.4 ng/mL [n = 20]) and at week 24 (mean 4.9 ng/mL [n = 6] versus 7.4 ng/mL [n = 11]), but patient numbers were low.

Multivariate exploratory analysis showed no association between the primary endpoint of the study (time to initiation of other anemia management or occurrence of Hb trigger) with hepcidin level at baseline (HR 0.92; 95% CI 0.73, 1.16; p = 0.49), the change in hepcidin from baseline to week 8 (HR 0.96; 95% CI 0.83, 1.11; p = 0.56) or the change to week 24 (HR 1.22; 95% CI 0.88, 1.68; p = 0.24) as covariates.

#### Association between hepcidin level and eGFR

There was no association between eGFR and hepcidin values at baseline (r = 0.22, p = 0.085), week 8 (r = 0.16, p = 0.24) or week 24 (r = 0.08, p = 0.61).

## Discussion

These are the first results from a prospective, randomized trial using defined iron therapy regimens to examine the three-way relationship between iron therapy, ferritin and hepcidin in patients with non-dialysis dependent CKD. Data over a one-year period show that hepcidin levels rise in response to iron therapy regardless of when the iron is administered periodically via IV administration or continuously via daily oral dosing. However, the speed and extent of the rise was greatest with IV iron targeting a higher ferritin level than with oral iron or when IV iron was used to target a lower ferritin level, reflecting differences in the rate of delivery of iron with each type of treatment.

Baseline hepcidin levels were within, or close to, the upper threshold for healthy controls in this population of non-dialysis dependent patients with CKD. This reflects the fact that this was an iron-deficient, relatively healthy cohort in which low levels of CRP indicated minimal chronic inflammation. At all time points there was a clear and significant correlation between serum hepcidin level and ferritin level. This is consistent with published data in hemodialysis patients [5, 6, 15, 18, 19, 27] and, as in our population, non-dialysis dependent patients with CKD [5, 20]. The utility of hepcidin as a marker for iron stores is limited, however, due to the high intra-individual variation in hepcidin levels [13]. An important regulator of hepcidin is inflammation, which stimulates production of hepcidin [3, 12], and chronic inflammation becomes most prominent as renal function declines [28]. Hepcidin levels correlate with the inflammatory marker C-reactive protein in patients on dialysis [13, 29, 30] or in non-dialysis dependent CKD [7, 20], and the wide intra-patient variability in hepcidin levels may reflect short-term fluctuations in the inflammatory state [13]. Thus, hepcidin levels may not be a reliable guide to whether iron therapy is required for patients with chronic inflammation, a situation which is already known to apply when ferritin is used as a biomarker for iron status.

In the complete cohort the correlation between hepcidin and TSAT levels was not significant, although the change from baseline to endpoint in both values did attain significance. However the window between iron administration and sampling may have influenced this result [21] since in the oral iron group, iron was administered daily whereas in the IV iron group iron was administered at least 4 weeks prior to blood sampling. Other authors have observed a significant positive association between hepcidin and TSAT in peritoneal dialysis [27] and non-dialysis dependent patients with CKD [20], but Ford *et al*, in their assessment of 28 hemodialysis patients, also found no significant correlation [13].

Neither baseline hepcidin levels nor change in hepcidin levels predicted the response to iron therapy whereas a very weak association between endpoint hepcidin levels and hemoglobin levels was found. Theoretically low baseline levels of hepcidin are associated with reduced systemic iron levels and increased capacity to absorb oral iron, and thus identify successful candidates for oral iron therapy. The effect of a marked increase in hepcidin on treatment response is harder to anticipate. If hepcidin is a marker of iron load and concurs with increased iron stores, increased levels are associated with successful therapy. However, if increased levels of hepcidin designate reduced absorption and reduced iron release from the iron stores a marked increase in hepcidin would be associated with resistance to therapy. In this study we observed no evidence that the latter contention is true. Overexpression of ferroportin in response to high iron levels may permit export of iron from macrophages into the circulation even when hepcidin levels are elevated [31]. In our analysis, there was no significant association between change in hepcidin levels and achievement of Hb thresholds or the primary endpoint of the study. Reports in the literature regarding a role for hepcidin in predicting response to therapy are relatively limited [10, 13, 16–20]. A single-arm study in 51 patients on hemodialysis with iron deficiency examined the association between hepcidin levels and response to oral iron therapy [18]. All patients had been receiving an ESA but no iron therapy for the three month prior to study entry, at which point oral ferrous fumarate 50 mg/ day was given for eight weeks. In the 16 patients who were Hb responders (i.e. Hb increase of 2 g/dL or higher), mean hepcidin was significantly lower than in non-responders (10.8 versus 32.8 ng/mL, p<0.05) [18]. Chand *et al* studied 129 patients with non-dialysis CKD given IV iron therapy, and on multivariable analysis observed hepcidin to be significantly predictive of Hb increase by week 8 after the start of iron infusion (p = 0.002) [20]. In contrast, Tessitore *et al* found baseline hepcidin levels to show no significant association with the response to IV iron [19]. In their series of 56 hemodialysis patients, multivariate analysis indicated that serum hepcidin level was not a significant predictor for an increase in Hb following IV iron therapy in addition to maintenance ESA therapy [19].

Research into the role of serum hepcidin as a biomarker has been hampered by accurate, consistent concentration measurements [32]. Different assay methods and a lack of standardization have made between-study comparisons difficult [33]. Absolute values for hepcidin in urine or plasma samples can vary by up to 10-fold between assay types and centers [34, 35]. Here, we used a validated liquid chromatography tandem mass spectrometry assay. This is more robust than immunoassays for detecting hepcidin (such as ELISA) which have been used in other investigative studies of hepcidin as a biomarker, and which cross-react with other hepcidin isoforms such as hepcidin-22 and hepcidin-20. The study also benefitted from its prospective, randomized design, and the extended 12-month longitudinal recording of hepcidin values. The population size was limited by the fact that only patients recruited in the UK took part in the substudy, but this ensured consistent measurement of hepcidin at a single center using a single assay. It should be noted that hepcidin values were excluded if patients reached the primary endpoint—which included initiation of ESA therapy—so these findings do not necessarily apply to CKD patients receiving ESAs. Similarly, baseline hepcidin values were low, indicating that the patient population did not have chronic inflammation (the study protocol excluded patients with active infection or baseline CRP ≥ 20 mg/L), limiting extrapolation to more inflamed patient groups. Lastly, we recognise that over the course of this one-year study hepcidin data were available in a declining number of patients, with fewer than half the hepcidin population providing values at one year. This effect was exacerbated by exclusion of patients in whom alternative anemia management was started. This avoided contamination of data regarding the relative effect of the randomized therapies, and effectively provided a 'per protocol analysis', but it also substantially reduced the data set over time.

This is the first analysis to prospectively assess the response of hepcidin levels to different iron treatments. Results from these pre-specified and *post ho*c analyses showed that baseline hepcidin levels were in the normal range, consistent with the presence of iron deficiency, anemia and low inflammatory state. With either IV or oral iron therapy, the increase in hepcidin correlated with the administered dose of iron. Changes in hepcidin levels showed a close correlation with the level of stored iron, as measured by ferritin, but not either TSAT or Hb levels. Hepcidin levels were not predictive for hematopoietic response to iron therapy in this population, although the size of this population and the increasing proportion of non-evaluable patients as the study progressed precludes firm conclusions. Further prospective studies, specifically in patients with chronic inflammation, are awaited.

## Supporting Information

S1 Appendix FIND-CKD trial: Ethics Committee approvals. (DOCX)

S1 Fig Proportion of patients receiving high dose FCM over time (FCM group, n = 154). (EPS)

S1 Table (DOCX)
