# Supplementary material for: Hepcidin Response to Iron Therapy in Patients with Non-Dialysis Dependent CKD: An Analysis of the FIND-CKD Trial
Source: PLoS One. 2016 Jun 8;11(6):e0157063. doi: 10.1371/journal.pone.0157063 (PMC4898697; doi:10.1371/journal.pone.0157063)
Supplement: S1 Appendix — (DOCX) [file pone.0157063.s001.docx]

**Appendix**

**FIND-CKD trial: Ethics Committee approvals**

| **Country** | **Site Nr** | **Ethic Committee** |  |
| --- | --- | --- | --- |
| Australia | 0101 | Bellberry HREC 229 Greenhill Road Dulwich SA 5O65 |  |
| Australia | 0102 | Bellberry HREC 229 Greenhill Road Dulwich SA 5O65 |  |
| Australia | 0103 | Hunter Area Research Ethics Committee John Hunter Hospital Lookout Road  New Lambton Heights NSW 23O5 |  |
| Australia | 0104 | Research Ethics Committee Royal Adelaide Hospital North Terrace  Adelaide SA 5OOO |  |
| Australia | 0105 | Human Research Ethics Committee Royal Melbourne Hospital Parkville, Victoria 3O5O |  |
| Australia | 0106 | Eastern Health HREC Level 2, 5 Arnold Street Box Hill, Victoria 3f28 |  |
| Australia | 0106 | Eastern Health HREC Level 2, 5 Arnold Street Box Hill, Victoria 3f29 |  |
| Australia | 0107 | Hunter Area Research Ethics Committee John Hunter Hospital Lookout Road  New Lambton Heights NSW 23O5 |  |
| Australia | 0108 | PAH Human Research Ethics Committee Tafe 3, Level 2, Bldg 35  Princess Alexandra Hospital Ispswich Road Woolloongabba, QLD 4fO2 |  |
| Australia | | 9 | Ballarat Health Services and St. John of God Health Care Ethics Committee  Base Hospital Drummond Street North PO Box 577 Ballarat 3353 |
| Australia | | 0110 | Hunter Area Research Ethics Committee John Hunter Hospital Lookout Road  New Lambton Heights NSW 2305 |
| Australia | | 0111 | Hunter Area Research Ethics Committee John Hunter Hospital Lookout Road  New Lambton Heights NSW 2306 |
| Australia | | 0111 | Royal Brisbane and Women's Hospital HREC University of Queensland, Centre for Clinical Research, Level 4, RBWH HERSTON, QUEENSLAND AUSTRALIA 4029 |
| Australia | | 0112 | Sir Charles Gairdner HREC Level 2, A block Hospital Avenue Nedlands, WA 6009 |
| Australia | | 0113 | Royal Perth Hospital HREC Colonial House Wellington Street, WA 6000 |
| Australia | | 0114 | Southern Health HREC 246 Clayton Road Clayton, Victoria 3168 |
| Australia | | 0115 | Sir Charles Gairdner HREC Level 2, A block Hospital Avenue Nedlands, WA 6009 |
| Australia | | 0117 | Cairns Base Hospital Ethics Committee PO Box 902 Cairns, QLD 4870 |
| Australia | | 0118 | Hunter Area Research Ethics Committee John Hunter Hospital Lookout Road  New Lambton Heights NSW 2305 |
| Australia | | 0119 | Hunter Area Research Ethics Committee John Hunter Hospital Lookout Road  New Lambton Heights NSW 2305 |
| Australia | | 0120 | Sir Charles Gairdner HREC Level 2, A block Hospital Avenue  Nedlands, WA 6009 |
| Austria | | 0202 | Ethikkommission der Stadt Wien Town Thomas-Klestil-Platz 8/2 A-1030 Wien, Osterreich |
| Austria | | 0203 | Ethikkommission der Stadt Wien Town Thomas-Klestil-Platz 8/2 A-1030 Wien, Osterreich |
| Austria | | 0204 | Ethikkommission des Landes Vorarlberg  Rathausstrassed 15  A-6900 Bregenz Osterreich |
| Austria | | 0205 | Ethikkommission Krankenhaus der Elisabethinen Linz GmbH Fadingerstrasse 1  A-4 Linz Osterreich |
| Austria | | 0206 | Ethikkommission der Medizinischen Universitat Innsbruck Innrain 43  A-6020 Innsbruck Osterreich |
| Austria | | 0207 | EK des Landes Oberosterreich Landesnervenklinik Wagner-Jauregg Strasse Wagner-Jauregg Weg 15  A-4020 Linz Osterreich |
| Belgium | | 0301 | Secretariaat Ethische Commissie UZ Gent Attn. Prof. Dr Matthys De Pintelaan 185  9000 Gent |
| Belgium | | 0302 | H.-Hartziekenhuis Roeselare-Menen vzw Attn. Dr. Ludo Marcelis WILGENSTRAAT 2  8800 ROESELARE |
| Belgium | | 0303 | Dr Van Vlem  Onze-Lieve-Vrouwziekenhuis Attn. Greet de Geest Moorselbaan 164 9300 Aalst |
| Belgium | | 0304 | Commissie Medische Ethiek van Universitaire Ziekenhuizen K.U.Leuven Attn. Prof. Walter Van den Bogaert  Campus Gasthuisberg E330 Herestraat 49 B-3000 Leuven |
| Belgium | | 0305 | Kristien Schoenmakers gang beheer en directie ZOL Campus St Jan Schiepse bos 6 3600 Genk |
| Belgium | | 0306 | Hopitaux IRIS Sud-site Joseph Bracops Rue Dr Huet 79 Brussels 1070 |
| Belgium | | 0307 | Comité d'Ethique du Epicura Ath-Baudour Attn. Dr Frederic Debelle  136 rue Louis Caty 7331 Baudour |
| Belgium | | 0308 | Commission d’Ethique Biomédicale Hospitalo- Facultaire Attn. Pr Jean-Marie Maloteaux Cliniques Universitaires Saint-Luc Avenue Hippocrate 55/14 B-1200 Bruxelles |
| Belgium | | 0309 | Comite d'Ethique Hospitalo-Facultaire Universitaire de Liege Centre Hospitalier Universitaire du Sart Tilman, B35 4000 Sart Tilman par Liege 1 |
| Belgium | | 0311 | Centre Hospitalier Universitaire Brugman Attn. Valsamis Joseph  Place A. Van Gehuchten, 4 1020 Bruxelles --2 |
| Belgium | | 0312 | Comite d'Ethique Clinique Universitaire de Bruxelles  Hopital Erasme Route de Lennik 808 1070 Bruxelles - 7 |
| Czech Republic | | 0401 | Etická komise IKEM a FN Thomayerovy s poliklinikou Vídeňská 800 140 59 Praha 4 |
| Czech Republic | | 0402 | Etická komise pro multicentrická hodnocení Fakultní nemocnice v Motole V Úvalu 84, 150 06 Praha 5 |
| Czech Republic | | 0403 | Etická komise Litomyšlská nemocnice  a.s. J. E. Purkyně 652 570 14 Litomyšl |
| Czech Republic | | 0404 | Etická komise Nemocnice Jihlava Vrchlického 59 586 01 Jihlava |
| Czech Republic | | 0405 | Etická komise pro multicentrická hodnocení Fakultní nemocnice v Motole V Úvalu 84, 150 06 Praha 5 |
| Czech Republic | | 0406 | Etická komise  Krajská nemocnice T. Bati a.s. Zlín Havlíčkovo nábřeží 600 762 75 Zlín |
| Czech Republic | | 0407 | Etická komise Fakultní nemocnice Hradec Králové Sokolská 581500 05 Hradec Králové |
| Czech Republic | | 0408 | Etická Komise Nemocnice Písek, a.s. Karla Čapka 589 397 23 Písek |
| Czech Republic | | 0409 | Etická komise Nemocnice Tábor, a.s. Kpt. Jaroše 2000 390 03 Tábor |
| Czech Republic | | 0410 | Etická komise  Nemocnice ve Frýdku-Místku, p.o. El. Krásnohorské 321 738 18 Frýdek-Místek |
| Czech Republic | | 0411 | Etická komise  FN Brno Bohunice Jihlavská 20 625 00 Brno |
| Czech Republic | | 0412 | Etická komise B. Braun Avitum Bulovka Budínova 67 181 02 Praha 8 |
| Czech Republic | | 0413 | Etická komise B. Braun Avitum Bulovka Budínova 67 181 02 Praha 8 |
| Czech Republic | | 0414 | Etická komise Nemocnice s poliklinikou v Novém Jičíně, p.o.  K Nemocnici 775/76 741 01 Nový Jičín |
| Czech Republic | | 0415 | Etická komise B. Braun Avitum Bulovka Budínova 67 181 02 Praha 8 |
| Czech Republic | | 0416 | Etická komise Nemocnice Znojmo MUDr. Jana Janského 11 669 02 Znojmo |
| Czech Republic | | 0417 | Etická komise B. Braun Avitum Bulovka Budínova 67 181 02 Praha 8 |
| Czech Republic | | 0418 | Etická komise společnosti Fresenius Medical Care - DS, s.r.o.  Lužná 591  160 05 Praha 6 |
| Czech Republic | | 0419 | Etická komise pro multicentrická hodnocení Fakultní nemocnice v Motole V Úvalu 84  150 06 Praha 5 |
| Czech Republic | | 0420 | Etická komise společnosti Fresenius Medical Care - DS, s.r.o.  Lužná 591  160 05 Praha 6 |
| Czech Republic | | 0421 | Etická komise společnosti Fresenius Medical Care - DS, s.r.o.  Lužná 591  160 05 Praha 6 |
| Czech Republic | | 0422 | Etická komise společnosti Fresenius Medical Care - DS, s.r.o.  Lužná 591  160 05 Praha 6 |
| Czech Republic | | 0423 | Etická komise společnosti Fresenius Medical Care - DS, s.r.o.  Lužná 591  160 05 Praha 6 |
| Czech Republic | | 0424 | Etická komise pro multicentrická hodnocení Fakultní nemocnice v Motole V Úvalu 84, 150 06 Praha 5 |
| Denmark | | 0501 | De Videnskabsetiske Komiteer for Region  Hovedstaden Regionsgarden Kongesn Vaenge  CK-3400 Hillerod |
| Denmark | | 0502 | De Videnskabsetiske Komiteer for Region  Hovedstaden Regionsgarden Kongesn Vaenge  CK-3400 Hillerod |
| Denmark | | 0503 | De Videnskabsetiske Komiteer for Region Hovedstaden  Regionsgården Kongens Vænge 2 DK-3400 Hillerød |
| Denmark | | 0504 | De Videnskabsetiske Komiteer for Region  Hovedstaden Regionsgarden Kongesn Vaenge  CK-3400 Hillerod |
| France | | 0601 | CPP Sud-Méditerranée IV  Dr Alain DUBOIS Hopital Saint Eloi  Rue Bertin Sand  34295 Montpellier  Cedex 5 |
| France | | 0601 | CPP Sud-Méditerranée IV  Dr Alain DUBOIS Hopital Saint Eloi  Rue Bertin Sand  34295 Montpellier  Cedex 5 |
| France | | 0602 | CPP Sud-Méditerranée IV Dr Alain DUBOIS Hôpital Saint Eloi Rue Bertin Sans 34295 Montpellier Cedex 5 |
| France | | 0603 | CPP Sud-Méditerranée IV Dr Alain DUBOIS Hôpital Saint Eloi Rue Bertin Sans 34295 Montpellier Cedex 5 |
| France | | 0604 | CPP Sud-Méditerranée IV Dr Alain DUBOIS Hôpital Saint Eloi Rue Bertin Sans 34295 Montpellier Cedex 5 |
| France | | 0605 | CPP Sud-Méditerranée IV Dr Alain DUBOIS Hôpital Saint Eloi Rue Bertin Sans 34295 Montpellier Cedex 5 |
| France | | 0606 | CPP Sud-Méditerranée IV Dr Alain DUBOIS Hôpital Saint Eloi Rue Bertin Sans 34295 Montpellier Cedex 5 |
| France | | 0607 | CPP Sud-Méditerranée IV Dr Alain DUBOIS Hôpital Saint Eloi Rue Bertin Sans 34295 Montpellier Cedex 5 |
| France | | 0608 | CPP Sud-Méditerranée IV Dr Alain DUBOIS Hôpital Saint Eloi Rue Bertin Sans 34295 Montpellier Cedex 5 |
| France | | 0609 | CPP Sud-Méditerranée IV Dr Alain DUBOIS Hôpital Saint Eloi Rue Bertin Sans 34295 Montpellier Cedex 5 |
| France | | 0610 | CPP Sud-Méditerranée IV Dr Alain DUBOIS Hôpital Saint Eloi Rue Bertin Sans 34295 Montpellier Cedex 5 |
| France | | 0611 | CPP Sud-Méditerranée IV Dr Alain DUBOIS Hôpital Saint Eloi Rue Bertin Sans 34295 Montpellier Cedex 5 |
| France | | 0612 | CPP Sud-Méditerranée IV Dr Alain DUBOIS Hôpital Saint Eloi Rue Bertin Sans 34295 Montpellier Cedex 5 |
| Germany | | 0701 | Ethik-Kommmission bei der Medizinischen Fakultät der Universitat Wurzburg Institut für Pharmakologie und Toxikologie Versbacher Str. 9  97078 Wurzburg Wuerzburg |
| Germany | | 0702 | Site 0702  Ethik-Kommission der Arztekammer Hamburg  Humboldtstr. 67a 22083 Hamburg |
| Germany | | 0703 | Site 0703  Wthikkommission an der Medizinischen Fakultat Ernst-Moritz-Arndt-Universitat Greifswald Institut fur Pharmakologie  Friedrich-Loeffler-Str. 23d 17487 Greifswald |
| Germany | | 0704 | Site 0704, 0721  Ethikkommission der Arztekammer Nordrhein Tersteegenstr. 9  40474 Dusseldorf |
| Germany | | 0705 | Site 0705, 0708, 0710, 0711  Ethikkommission der Arztekammer Westfalen-Lippe und der Medizinischen Fakultat der WWU-Munster Von-Esmarch-Strasse 62  48149 Munster |
| Germany | | 0706 | Site 0706  Ethikkommission der Universitat Ulm Helmholtzstrasse 20  (Oberer Eselsberg) 89081 Ulm |
| Germany | | 0708 | Site 0705, 0708, 0710, 0711  Ethikkommission der Arztekammer Westfalen-Lippe und der Medizinischen Fakultat der WWU-Munster Von-Esmarch-Strasse 62  48149 Munster |
| Germany | | 0709 | Site 0709, 0729  Ethik-Kommission der Landesarztekammer Hessen Im Vogelsang 3  60488 Frankfurt am Main |
| Germany | | 0710 | Site 0705, 0708, 0710, 0711  Ethikkommission der Arztekammer Westfalen-Lippe und der Medizinischen Fakultat der WWU-Munster Von-Esmarch-Strasse 62  48149 Munster |
| Germany | | 0711 | Site 0705, 0708, 0710, 0711  Ethikkommission der Arztekammer Westfalen-Lippe und der Medizinischen Fakultat der WWU-Munster Von-Esmarch-Strasse 62  48149 Munster |
| Germany | | 0712 | Site 0712, 0726  Ethik-Kommission der Bayerischen Landesarztekammer Muhlbaurstrasse 16 81677 Munchen |
| Germany | | 0713 | Site 0713, 0725  Landesamt fur Gesundheit und Soziales Geschaftsstelle der Ethik-Kommission des Landes Berlin Fehrbelliner Platz 1  10707 Berlin |
| Germany | | 0714 | Site 0714, 0722  Ethikkommission Landesarztekammer Rheinland-Pfalz Deutschhausplatz 3 55116 Mainz |
| Germany | | 0715 | Site 0715  Ethikkommission an der Med. Fakultat der Rheinischen Friedrich-Wilhelms-Universitat Bonn  Biomedizinisches Zentrum Sigmund-Freud-Str. 25 53105 Bonn |
| Germany | | 0716 | Site 0716  Ethik-Kommission des Fachbereichs Medizin der Johann Wolfgang Goethe- Universitat Haus 1 Theodor-Stern-Kai 7  60590 Frankfurt |
| Germany | | 0717 | Site 0717, 0730  Ethik-Kommission bei der Landesarztekammer Baden-Wurttemberg Jahnstr. 40 70597 Stuttgart |
| Germany | | 0719 | Site 0719  Ethikkommission bei der Arztekammer Niedersachsen zur Beurteilung Medizinischer  Forschung am Menschen Berliner Allee 20 30175 Hannover |
| Germany | | 0720 | Site 0720  Ethikkommission bei der Sachsischen Landesarztekammer Schutzenhohe 16  99 Dresden |
| Germany | | 0721 | Site 0704, 0721  Ethikkommission der Arztekammer Nordrhein Tersteegenstr. 9  40474 Dusseldorf |
| Germany | | 0722 | Site 0714, 0722  Ethikkommission Landesarztekammer Rheinland-Pfalz Deutschhausplatz 3 55116 Mainz |
| Germany | | 0724 | Site 0724  Ethik-Kommission der Otto-von-Guericke- Universitat an der Medizinischen Fakultat Leipziger Str. 44 39120 Magdeburg |
| Germany | | 0725 | Site 0713, 0725  Landesamt fur Gesundheit und Soziales Geschaftsstelle der Ethik-Kommission des Landes Berlin Fehrbelliner Platz 1  10707 Berlin |
| Germany | | 0726 | Site 0712, 0726  Ethik-Kommission der Bayerischen  Landesarztekammer  Muhlbaurstrasse 16  81677 Munchen |
| Germany | | 0727 | Site 0727  Ethik-Kommission der Universitat Witten/Herdecke Alfred-Herrhausen-Str. 50  58448 Witten |
| Germany | | 0728 | Site 0728 |
|  |  |  | Ethikkommission der Med. Fakultat der |
|  |  |  | Universitat zu Koln |
|  |  |  | Gebaude 5 |
|  |  |  | Kerpener Str. 62 |
|  |  |  | 50937 Koln |
| Germany | | 0729 | Site 0709, 0729 |
|  |  |  | Ethik-Kommission der Landesarztekammer |
|  |  |  | Hessen |
|  |  |  | Im Vogelsang 3 |
|  |  |  | 60488 Frankfurt am Main |
| Germany | | 0730 | Site 0717, 0730 |
|  |  |  | Ethik-Kommission bei der |
|  |  |  | Landesarztekammer Baden-Wurttemberg |
|  |  |  | Jahnstr. 40 |
|  |  |  | 70597 Stuttgart |
| Germany | | 0731 | Site 0731 |
|  |  |  | Ethik-Kommission der Landesarztekammer |
|  |  |  | Brandenburg |
|  |  |  | Dreifertstrasse 12 |
|  |  |  | 03044 Cottbus |
| Greece | | 0801 | Ethical Committee  General University Hospital of Thessaloniki "Papgeorgiou" Thessaloniki Ring Road, Nea Efkarpia Thessaloniki, 56429 |
| Greece | | 0802 | General Hospital of Thessaloniki "Ippokrateion" 49 Konstantinoupoleos st.  Thessaloniki, 56442 |
| Greece | | 0803 | ATTIKON General University Hospital of Athens 1 Rimini Str.  Chaidari, Athens, 12462 |
| Greece | | 0804 | General University Hospital of Alexandroupolis Dragana Alexandroupolis, 68100 |
| Greece | | 0805 | Ethical Commitee  General University Hospital of Larissa Mezourlo Larissa, 41110 |
| Greece | | 0806 | Ethics Committee  Achillopoulio General Prefecture Hospital of Volos 134 Polyeri street  Volos, 38222 |
| Greece | | 0807 | Ethical Commitee  General Hospital of Rhodes Aghioi Aphostoloi Rhodes, 85100 |
| Greece | | 0808 | Ethical Commitee  IPPOKRATEION General Hospital of Athens 114 Vas. Sofias Ave Athens, 11526 |
| Greece | | 0809 | Ethical Commitee  General Hospital of Athens, KORGALENEIO- BENAKEIO Athenasaki Str. 1  Athens, 11526 |
| Greece | | 0810 | Ethical Commitee  General Prefecture Hospital of Ioannina, XATZIKOSTA Avv. Makrigianni 1  Ioannina, 45550 |
| Greece | | 0811 | Ethical Commitee  General University Hospital of Patras Rio-Patras Street Rios Patras, 16500 |
| Greece | | 0812 | Ethical Commitee  General University Hospital of Ioannina Stavros Niarchos Avenue Ioannina, 45550 |
| Greece | | 0813 | Ethical Commitee General Hospital of Arta   1. Zara Str 4 Arta, 47100 |
| Greece | | 0815 | Ethical Commitee  General Hospital of Peireus "Tzaneio" Zanni & Afendouli Peireus, 18536 |
| Greece | | 0817 | Ethical Commitee  General Prefecture Hospital of Argos 191 Korinthou Str.  Argos, 21200 |
| Greece | | 0818 | Ethical Commitee  LAIKO General Hospital of Athens 17 Aghiou Thomas Str.  Athens, 11527 |
| Greece | | 0819 | Ethical Commitee  General Hospital of Mytilene "Vostanio" 48 E. Vostani Str.  Vestos, 81100 |
| Greece | | 0820 | Ethical Commitee General Hospital of Serres 2nd k of Serres-Drama National Road Serres, 62100 |
| Greece | | 0821 | Ethical Commitee  KYANOUS STAVROS General Hospital of Athens  102, Vas Sofias Ave Athens, 11528 |
| Greece | | 0822 | Ethical Commitee  IASO General Hospital of Athens Cholargos Athens, 11526 |
| Greece | | 0823 | Ethical Commitee  General Hospital of Athens "Henry Dunant" 107 Messogheion Ave Athens, 11526 |
| Italy | | 1001 | Comitato Etico  Dell'Azienda Ospedaliera di Lecco Via Dell'Eremo 9/11 Lecco, 23900 |
| Italy | | 1002 | Comitato Etico Locale per la Sperimentazione Clinical Della AUSL 12 di Viareggio Via Aurelia 335  55045 Lido di Cà Maiore (LC) |
| Italy | | 1003 | Comitato Etico Dell'Azeinda Ospedaliera Universitaria Della Seconda Univestità degli Studi di Napoli Via Costatinopoli, 104  80138 Napoli |
| Italy | | 1004 | Comitato Etico Indipendente dell'Azienda ospedaliero-Univesitaria Policlinico S. Orsola Via Albertoni 15  40138 Bologna |
| Italy | | 1005 | Comitato Etico Della ASL TO/2 di Torino Corso Svizzera 185 bis 10149 Torino |
| Italy | | 1007 | Comitato di Bioetica della Azienda Ospedali Riuniti di Bergam Largo Barozzi 1  24128 Bergamo |
| Italy | | 1008 | Comitato Etico ASL di Caserta Via Unità Italiana 28 81100 Caserta |
| Italy | | 1009 | Comitato Etico Dell'Azienda Ospedaliera Universitaria 'S. Martin' di Genova Largo Rosanna Benzi 10 16132 Genova |
| Italy | | 1 | Comitato Etico Della Provincia di Modena Via Largo del Pozzo 71 41124 Modena |
| Italy | | 1011 | Comitato Etico ASL CE/1 Di Caserta Via Unità Italiana 28 81100 Caserta |
| Italy | | 1012 | Comitato Etico Regionale Unico (CERU) AOU Santa Maria della Misericordia Piazzale Santa maria della Misericordia 15 33100 Udine |
| Italy | | 1013 | Comitato Etico Scientifico Dell'Azienda Ospedaliera Ospedale S. Carlo Borromeo di Milano  Via Pio II° n° 3 20153 Milano |
| Italy | | 1014 | Comitato Bioetico Dell'Azienda Cannizzaro di Catania Via Messina 829  95126 Catania |
| Italy | | 1016 | Comitato Ethico-Scientifico Dell'Azienda Ospedaliera Ospedale Niguara Ca' Granda Di Milano Piazza Ospedale Maggiore n. 3 20162 Milano |
| Italy | | 1017 | Comitato Etico Della AUSL RM/H Di Albano Laziale  Borgo Garibaldi n. 12 00041 Albano Laziale (RM) |
| Italy | | 1018 | Comitato Etico Dell'Azienda Ospedaliera Pugliese-Ciaccio Di Catanzaro  Via Vinicio Cortese, 10 88100 Catanzaro |
| Italy | | 1020 | Comitato Etico Dell'Azienda Ospedaliera Universitaria S. Giovanni Battista di Torino C so Bramante 88/90 10126 Torino |
| Italy | | 1021 | Comitato Etico Scientifico Dell'Azienda Ospedaliera Ospedale S. Carlo Borrome di Milano  Via Pio II°, n°3 20153 Milano |
| Italy | | 1022 | Comitato Etico Central Dell'IRCCS Fondazione Salvatore Maugeri Di Pavia Via Salvatore Maugeri 4 27100 Pavia |
| Italy | | 1023 | Comitato Etico Sperimentazione clinical Medicinali Della AUSL 8 Di Arezzo Via Curtatone 54  52100 Arezzo |
| Italy | | 1024 | Comitato Etico  Azienda Ospedaliera Universitaria Ospedali Riuniti di figgia  Viale Luigi Pinto 71100 Foggia |
| Italy | | 1026 | Comitato di Etica Della ASL di Salerno Via Federico Ricco, 50 84014 Noceria Inferiore (SA) |
| Italy | | 1027 | Comitato Etico Per le Sperimentazioni Cliniche die Medicinali Della Provincia di Venezia  Via Don Federico Tosatto 147 30174 Venezia |
| Italy | | 1028 | Comitato Etico Della AUSL RM/G di Tivoli Via Tiburtina 22/a 00019 Tivoli (RM) |
| Italy | | 1029 | Comitato Etico Delle Aziende Sanitarie Dell'Umbria di Perugia  Via della Rivoluzione 16 Ellera di Corciano (PG) 06070 Perugia |
| Netherlan ds | | 1101 | Meander Medical Center, Lichtenberg location Toetsingscommissie Wetenschappelijk Onderzoek  Secretariat, P&O Room N042 Utrechtseweg 160  3818 ES Amersfoort The Netherlands |
| Netherlan ds | | 1102 | Medical Ethics Review Committee Zuidwest Holland Fonteynenburghlaan 7  2275 CX VOORBURG  The Netherlands |
| Netherlan ds | | 1102 | Medical Ethics Review Committee Gelre Hospital Albert Schweitzerlaan 31  7334 DZ Apeldoorn The Netherlands |
| Netherlan ds | | 1103 | Medical Ethics Review Committee Gelre Hospital Albert Schweitzerlaan 31  7334 DZ Apeldoorn The Netherlands |
| Netherlan ds | | 1104 | Medical Ethics Review Committee Albert Schweitzer Hospital loc. DW, Postvak 7, kmr. Z 150  T.a.v. Ms. A. de Graag – de Vries Albert Schweitzerplaats 25 3318 AT Dordrecht The Netherlands |
| Netherlan ds | | 1105 | METc VU Medical Center Medical Faculty, Room H-565 Van der Boerchorststraat 7 1081 BT Amsterdam The Netherlands |
| Netherlan ds | | 1106 | Medical Ethics Review Committee Noord- Holland  Foreest Medical School Nassauplein 10 1815 GM Alkmaar The Netherlands |
| Norway | | 1201 | Regional Committees for Medical and Health Research Ethics (REK) REK-Midt  Bygg for samfunnsmedisin (5 etg) Håkon Jarlsgt. 11, St. Olavs Hospital Trondheim |
| Poland | | 1301 | Niezależna Komisja Bioetyczna do Spraw Badań Naukowych przy Gdańskim Uniwersytecie Medycznym ul. M. Skłodowskiej-Curie 3a, 80-201 Gdańsk, Polska |
| Poland | | 1302 | Niezależna Komisja Bioetyczna do Spraw Badań Naukowych przy Gdańskim Uniwersytecie Medycznym ul. M. Skłodowskiej-Curie 3a, 80-201 Gdańsk, Polska |
| Poland | | 1303 | Niezależna Komisja Bioetyczna do Spraw Badań Naukowych przy Gdańskim Uniwersytecie Medycznym ul. M. Skłodowskiej-Curie 3a, 80-201 Gdańsk, Polska |
| Poland | | 1306 | Niezależna Komisja Bioetyczna do Spraw Badań Naukowych przy Gdańskim Uniwersytecie Medycznym ul. M. Skłodowskiej-Curie 3a, 80-201 Gdańsk, Polska |
| Poland | | 1309 | Niezależna Komisja Bioetyczna do Spraw Badań Naukowych przy Gdańskim Uniwersytecie Medycznym ul. M. Skłodowskiej-Curie 3a, 80-201 Gdańsk, Polska |
| Poland | | 1311 | Niezależna Komisja Bioetyczna do Spraw Badań Naukowych przy Gdańskim Uniwersytecie Medycznym ul. M. Skłodowskiej-Curie 3a, 80-201 Gdańsk, Polska |
| Poland | | 1313 | Niezależna Komisja Bioetyczna do Spraw Badań Naukowych przy Gdańskim Uniwersytecie Medycznym ul. M. Skłodowskiej-Curie 3a, 80-201 Gdańsk, Polska |
| Poland | | 1314 | Niezależna Komisja Bioetyczna do Spraw Badań Naukowych przy Gdańskim Uniwersytecie Medycznym ul. M. Skłodowskiej-Curie 3a, 80-201 Gdańsk, Polska |
| Poland | | 1315 | Niezależna Komisja Bioetyczna do Spraw Badań Naukowych przy Gdańskim Uniwersytecie Medycznym ul. M. Skłodowskiej-Curie 3a, 80-201 Gdańsk, Polska |
| Poland | | 1316 | Niezależna Komisja Bioetyczna do Spraw Badań Naukowych przy Gdańskim Uniwersytecie Medycznym ul. M. Skłodowskiej-Curie 3a, 80-201 Gdańsk, Polska |
| Poland | | 1318 | Niezależna Komisja Bioetyczna do Spraw Badań Naukowych przy Gdańskim Uniwersytecie Medycznym ul. M. Skłodowskiej-Curie 3a, 80-201 Gdańsk, Polska |
| Poland | | 1320 | Niezależna Komisja Bioetyczna do Spraw Badań Naukowych przy Gdańskim Uniwersytecie Medycznym ul. M. Skłodowskiej-Curie 3a, 80-201 Gdańsk, Polska |
| Poland | | 1321 | Niezależna Komisja Bioetyczna do Spraw Badań Naukowych przy Gdańskim Uniwersytecie Medycznym ul. M. Skłodowskiej-Curie 3a, 80-201 Gdańsk, Polska |
| Poland | | 1322 | Niezależna Komisja Bioetyczna do Spraw Badań Naukowych przy Gdańskim Uniwersytecie Medycznym ul. M. Skłodowskiej-Curie 3a, 80-201 Gdańsk, Polska |
| Poland | | 1323 | Niezależna Komisja Bioetyczna do Spraw Badań Naukowych przy Gdańskim Uniwersytecie Medycznym ul. M. Skłodowskiej-Curie 3a, 80-201 Gdańsk, Polska |
| Poland | | 1324 | Niezależna Komisja Bioetyczna do Spraw Badań Naukowych przy Gdańskim Uniwersytecie Medycznym ul. M. Skłodowskiej-Curie 3a, 80-201 Gdańsk, Polska |
| Poland | | 1326 | Niezależna Komisja Bioetyczna do Spraw Badań Naukowych przy Gdańskim Uniwersytecie Medycznym ul. M. Skłodowskiej-Curie 3a, 80-201 Gdańsk, Polska |
| Poland | | 1327 | Niezależna Komisja Bioetyczna do Spraw Badań Naukowych przy Gdańskim Uniwersytecie Medycznym ul. M. Skłodowskiej-Curie 3a, 80-201 Gdańsk, Polska |
| Poland | | 1328 | Niezależna Komisja Bioetyczna do Spraw Badań Naukowych przy Gdańskim Uniwersytecie Medycznym ul. M. Skłodowskiej-Curie 3a, 80-201 Gdańsk, Polska |
| Poland | | 1329 | Niezależna Komisja Bioetyczna do Spraw Badań Naukowych przy Gdańskim Uniwersytecie Medycznym ul. M. Skłodowskiej-Curie 3a, 80-201 Gdańsk, Polska |
| Poland | | 1330 | Niezależna Komisja Bioetyczna do Spraw Badań Naukowych przy Gdańskim Uniwersytecie Medycznym ul. M. Skłodowskiej-Curie 3a, 80-201 Gdańsk, Polska |
| Poland | | 1331 | Niezależna Komisja Bioetyczna do Spraw Badań Naukowych przy Gdańskim Uniwersytecie Medycznym ul. M. Skłodowskiej-Curie 3a, 80-201 Gdańsk, Polska |
| Portugal | | 1402 | CEIC- National Ethics Committee for Clinical Investigation Parque da Saúde de Lisboa- Avenida do Brasil, 53 1749-004 Lisboa- Portugal |
| Portugal | | 1403 | CEIC- National Ethics Committee for Clinical Investigation Parque da Saúde de Lisboa- Avenida do Brasil, 53 1749-004 Lisboa- Portugal |
| Portugal | | 1404 | CEIC- National Ethics Committee for Clinical Investigation Parque da Saúde de Lisboa- Avenida do Brasil, 53 1749-004 Lisboa- Portugal |
| Portugal | | 1405 | CEIC- National Ethics Committee for Clinical Investigation Parque da Saúde de Lisboa- Avenida do Brasil, 53 1749-004 Lisboa- Portugal |
| Portugal | | 1406 | CEIC- National Ethics Committee for Clinical Investigation Parque da Saúde de Lisboa- Avenida do Brasil, 53 1749-004 Lisboa- Portugal |
| Portugal | | 1407 | CEIC- National Ethics Committee for Clinical Investigation Parque da Saúde de Lisboa- Avenida do Brasil, 53 1749-004 Lisboa- Portugal |
| Spain | | 1501 | Hospital Universitario Dr Peset de Valencia CEIC, a/a Raquel E. Blesa, C/Juan de Garray 21, 1er Piso Consultas externas, 46017 Valencia |
| Spain | | 1503 | Hospital Universitario Dr Peset de Valencia CEIC, a/a Raquel E. Blesa, C/Juan de Garray 21, 1er Piso Consultas externas, 46017 Valencia |
| Spain | | 1504 | CEIC Hospital Universitario La Paz(LEC) Paseo de la Castellana, 261, Planta 8a Hospital General, 28046 Madrid |
| Spain | | 1505 | CEIC Fundació Puigvert IUNA (LEC) Agencia de Gestio del Coneixement Cartagena, 340-350 08025 Barcelona |
| Spain | | 1506 | CEIC Hospital Universitario General Gregorio Marañón (CEC) CEIC Area 1, C/ dr Esquerdo, 46, 28007 Madrid |
| Spain | | 1507 | Agencia de Ensayos Clinicos - servicio de Farmacia Hospital Clinic de Barcelona, c/ Villarroel, 170 - Sotano, Escalera 6b, 08036 Barcelona |
| Spain | | 1509 | CEIC Hospital Universitario de Bellvitge Edificio Consultas Externas. Planta -1, C/ Feixa Llarga, s/n, 08907 L'Hospitalet de Llobregat, Barcelona |
| Spain | | 1510 | CEIC Hospital Universitari Vall d'Hebron Edifici Institut de Recerca, 2a planta Passeig Vall d'Hebron 119-129, Barcelona 08035 |
| Spain | | 1512 | CEIC Hospital Universitario Fundación de Alcorcón (LEC) C/ Budapest N1, 28922 Alcorcon, Madrid |
| Spain | | 1513 | CEIC Clinica de Asturias  Hospital Central de Asturias, Celestino Villamil, s/n, 33006 Oviedo |
| Spain | | 1514 | Hopsital Universitario "Reina Sofia" Comite Etico de Ensayos Clinicos, Edificio de Consultas Externas, planta -1, Avda. Menendez Pidal, s/n, 14004 Cordoba |
| Spain | | 1515 | Hospital Torrecardenas CEIC  Paraje Torrecardenas, s/n, 04009 Almeria |
| Spain | | 1516 | Fundacion Jimenez Diaz CEIC, Avda. Reyes Catolicos, 2, Entrplanta, 28040 Madrid |
| Spain | | 1517 | Hospital Universitario Principe de Asturias CEIC, Ctra. Alcala-Meco s/n, 28805 Alcala de Henares, Madrid |
| Spain | | 1518 | CEIC de Aragon, Avda. Gomez Laguna, 25 planta 11, 50009 Zaragoza |
| Spain | | 1519 | Hospital Universitario de Puerto Real, Ctra. NaI IV, km. 665, 11510 Puerto Real, Cadiz |
| Spain | | 1520 | CEIC Hospital Universitario de Getafe (LEC) Ctra. De Toledo, km. 12500, 28905 Getafe, Madrid |
| Spain | | 1521 | CEIC Hospital Universitario La Princesa, Findacion para la Investigacion Biomedica, C/ Diego de leon, 62, 28006 Madrid |
| Spain | | 1522 | CEIC Hospital Universitario de Girona Josep Trueta (LEC) avda. De Franca s/n, 17007 Girona |
| Spain | | 1523 | CEIC Parc Salut del Mar (LEC)  IMIM-Hospital del Mar, Parc de Recerca Biomedica de Barcelona, Doctor Aiguader, 88, 08003 Barcelona |
| Sweden | | 1601 | Regionala etikprövningsnämnden Stockholm FE 289  Karolinska Institutet  Stockholm, 17179 |
| Sweden | | 1602 | Regionala etikprövningsnämnden Stockholm FE 289  Karolinska Institutet  Stockholm, 17179 |
| Sweden | | 1603 | Regionala etikprövningsnämnden Stockholm FE 289  Karolinska Institutet  Stockholm, 17179 |
| Switzerlan d | | 1701 | Kantonal Ethikkommission Aargau Departement Gesundheit und Soziales PD Dr. med. Otto Hilfiker Bachstrasse 15  5001 Aarau |
| Switzerlan d | | 1702 | Kantonal Ethik-Kommission (KEK) Prof. Dr. med. Robert Maurer Universitätsspital Zürich Sonneggstr. 12 8091 Zürich |
| Switzerlan d | | 1703 | Kantonal Ethik-Kommission (KEK) Prof. Dr. med. Robert Maurer Universitätsspital Zürich Sonneggstr. 12 8091 Zürich |
| Switzerlan d | | 1704 | Kantonal Ethikkommission Bern (KEK) Prof. Dr. pharm. Nilaus Tüller Postfach 56 3 Bern |
| Turkey | | 1801 | Ankara University Medical Faculty Deanship Clinical Researches Ethics Committee nkara Universitesi Tip Fakultesi Morfoloji Binası 06100 Sihhiye Ankara Turkey |
| Turkey | | 1802 | Ege University Medical Faculty Clinical Researches Ethics Committee Ege Universitesi Tip Fakultesi; 35100 Bornova Izmir |
| Turkey | | 1803 | Ege University Medical Faculty Clinical Researches Ethics Committee Ege Universitesi Tip Fakultesi; 35100 Bornova Izmir |
| Turkey | | 1804 | Ege University Medical Faculty Clinical Researches Ethics Committee Ege Universitesi Tip Fakultesi; 35100 Bornova Izmir |
| Turkey | | 1804 | Ege University Medical Faculty Clinical Researches Ethics Committee Ege Universitesi Tip Fakultesi; 35100 Bornova Izmir |
| Turkey | | 1807 | Ege University Medical Faculty Clinical Researches Ethics Committee Ege Universitesi Tip Fakultesi; 35100 Bornova Izmir |
| Turkey | | 1810 | Ege University Medical Faculty Clinical Researches Ethics Committee Ege Universitesi Tip Fakultesi; 35100 Bornova Izmir |
| Turkey | | 1811 | Ege University Medical Faculty Clinical Researches Ethics Committee Ege Universitesi Tip Fakultesi; 35100 Bornova Izmir |
| United Kingdom | | 1901 | Health Research Authority NRES Committee Riverside REC Bristol REC Centre  Level 3, Block B Whitefriars Lewins Mead Bristol, BS 1 2NT |
| United Kingdom | | 1902 | Health Research Authority NRES Committee Riverside REC Bristol REC Centre  Level 3, Block B Whitefriars Lewins Mead Bristol, BS 1 2NT |
| United Kingdom | | 1903 | Health Research Authority NRES Committee Riverside REC Bristol REC Centre  Level 3, Block B Whitefriars Lewins Mead Bristol, BS 1 2NT |
| United Kingdom | | 1904 | Health Research Authority NRES Committee Riverside REC Bristol REC Centre  Level 3, Block B Whitefriars Lewins Mead Bristol, BS 1 2NT |
| United Kingdom | | 1905 | Health Research Authority NRES Committee Riverside REC Bristol REC Centre  Level 3, Block B Whitefriars Lewins Mead Bristol, BS 1 2NT |
| United Kingdom | | 1906 | Health Research Authority  NRES Committee Riverside REC  Bristol REC Centre  Whitefriars, Lewins Mead |
| United Kingdom | | 1907 | Health Research Authority  NRES Committee Riverside REC  Bristol REC Centre  Whitefriars, Lewins Mead |
| United Kingdom | | 1908 | Health Research Authority  NRES Committee Riverside REC  Bristol REC Centre  Whitefriars, Lewins Mead |
| United Kingdom | | 1909 | Health Research Authority  NRES Committee Riverside REC  Bristol REC Centre  Whitefriars, Lewins Mead |
| United Kingdom | | 1910 | Health Research Authority  NRES Committee Riverside REC  Bristol REC Centre  Whitefriars, Lewins Mead |
| United Kingdom | | 1911 | Health Research Authority  NRES Committee Riverside REC  Bristol REC Centre  Whitefriars, Lewins Mead |
| United Kingdom | | 1912 | Health Research Authority  NRES Committee Riverside REC  Bristol REC Centre  Whitefriars, Lewins Mead |
| United Kingdom | | 1913 | Health Research Authority  NRES Committee Riverside REC  Bristol REC Centre  Whitefriars, Lewins Mead |
| United Kingdom | | 1914 | Health Research Authority  NRES Committee Riverside REC  Bristol REC Centre  Whitefriars, Lewins Mead |
| United Kingdom | | 1915 | Health Research Authority  NRES Committee Riverside REC  Bristol REC Centre  Whitefriars, Lewins Mead |
| United Kingdom | | 1916 | Health Research Authority  NRES Committee Riverside REC  Bristol REC Centre  Whitefriars, Lewins Mead |
| United Kingdom | | 1917 | Health Research Authority NRES Committee Riverside REC Bristol REC Centre  Level 3, Block B Whitefriars Lewins Mead Bristol, BS 1 2NT |
| United Kingdom | | 1918 | Health Research Authority NRES Committee Riverside REC Bristol REC Centre  Level 3, Block B Whitefriars Lewins Mead Bristol, BS 1 2NT |
| United Kingdom | | 1919 | Health Research Authority NRES Committee Riverside REC Bristol REC Centre  Level 3, Block B Whitefriars Lewins Mead Bristol, BS 1 2NT |
| United Kingdom | | 1920 | Health Research Authority NRES Committee Riverside REC Bristol REC Centre  Level 3, Block B Whitefriars Lewins Mead Bristol, BS 1 2NT |
| United Kingdom | | 1921 | Health Research Authority NRES Committee Riverside REC Bristol REC Centre  Level 3, Block B Whitefriars Lewins Mead Bristol, BS 1 2NT |
| United Kingdom | | 1922 | Health Research Authority NRES Committee Riverside REC Bristol REC Centre  Level 3, Block B Whitefriars Lewins Mead Bristol, BS 1 2NT |
| United Kingdom | | 1923 | Health Research Authority NRES Committee Riverside REC Bristol REC Centre  Level 3, Block B Whitefriars Lewins Mead Bristol, BS 1 2NT |
| United Kingdom | | 1924 | Health Research Authority NRES Committee Riverside REC Bristol REC Centre  Level 3, Block B Whitefriars Lewins Mead Bristol, BS 1 2NT |
| United Kingdom | | 1925 | Health Research Authority NRES Committee Riverside REC Bristol REC Centre  Level 3, Block B Whitefriars Lewins Mead Bristol, BS 1 2NT |
| United Kingdom | | 1926 | Health Research Authority NRES Committee Riverside REC Bristol REC Centre  Level 3, Block B Whitefriars Lewins Mead Bristol, BS 1 2NT |
| United Kingdom | | 1927 | Health Research Authority NRES Committee Riverside REC Bristol REC Centre  Level 3, Block B Whitefriars Lewins Mead Bristol, BS 1 2NT |
| United Kingdom | | 1928 | Health Research Authority NRES Committee Riverside REC Bristol REC Centre  Level 3, Block B Whitefriars Lewins Mead Bristol, BS 1 2NT |
| United Kingdom | | 1929 | Health Research Authority NRES Committee Riverside REC Bristol REC Centre  Level 3, Block B Whitefriars Lewins Mead Bristol, BS 1 2NT |
| United Kingdom | | 19230 | Health Research Authority NRES Committee Riverside REC Bristol REC Centre  Level 3, Block B Whitefriars Lewins Mead Bristol, BS 1 2NT |
| United Kingdom | | 1931 | Health Research Authority NRES Committee Riverside REC Bristol REC Centre  Level 3, Block B Whitefriars Lewins Mead Bristol, BS 1 2NT |
| United Kingdom | | 1932 | Health Research Authority NRES Committee Riverside REC Bristol REC Centre  Level 3, Block B Whitefriars Lewins Mead Bristol, BS 1 2NT |
| United Kingdom | | 1933 | Health Research Authority NRES Committee Riverside REC Bristol REC Centre  Level 3, Block B Whitefriars Lewins Mead Bristol, BS 1 2NT |
| Romania | | 2001 | National Ethics Committee for Clinical Study of Medicine (Comisia Nationala de Etica pentru Studiul Clinic al Medicamentului) Av. Sanatescu St., No. 48, 1st district, 011478, Bucharest, Romania |
| Romania | | 2002 | National Ethics Committee for Clinical Study of Medicine (Comisia Nationala de Etica pentru Studiul Clinic al Medicamentului) Av. Sanatescu St., No. 48, 1st district, 011478, Bucharest, Romania |
| Romania | | 2003 | National Ethics Committee for Clinical Study of Medicine (Comisia Nationala de Etica pentru Studiul Clinic al Medicamentului) Av. Sanatescu St., No. 48, 1st district, 011478, Bucharest, Romania |
| Romania | | 2004 | National Ethics Committee for Clinical Study of Medicine (Comisia Nationala de Etica pentru Studiul Clinic al Medicamentului) Av. Sanatescu St., No. 48, 1st district, 011478, Bucharest, Romania |
| Romania | | 2005 | National Ethics Committee for Clinical Study of Medicine (Comisia Nationala de Etica pentru Studiul Clinic al Medicamentului) Av. Sanatescu St., No. 48, 1st district, 011478, Bucharest, Romania |
| Romania | | 2006 | National Ethics Committee for Clinical Study of Medicine (Comisia Nationala de Etica pentru Studiul Clinic al Medicamentului) Av. Sanatescu St., No. 48, 1st district, 011478, Bucharest, Romania |
| Romania | | 2007 | National Ethics Committee for Clinical Study of Medicine (Comisia Nationala de Etica pentru Studiul Clinic al Medicamentului) Av. Sanatescu St., No. 48, 1st district, 011478, Bucharest, Romania |
| US | | 2110* | Salem VA Medical Center IRB Kim Ragsdale, PhD 1970 Roanoke Blvd  Salem. VA 24153 |
| US | | 2113 | Integreview IRB Valerie Nelson  3001, South Lamar Blvd Suite 210 Austin, TX 78704 |
| US | | 2105 | Integreview IRB Valerie Nelson  3001, South Lamar Blvd Suite 210 Austin, TX 78704 |
| US | | 2114 | Temple VA Medical Cener IRB John W Klocek, PhD 1901 Veterans Memorial Drive Temple, TX 76504 |
