# Supplementary material for: Hepcidin Response to Iron Therapy in Patients with Non-Dialysis Dependent CKD: An Analysis of the FIND-CKD Trial
Source: PLoS One. 2016 Jun 8;11(6):e0157063. doi: 10.1371/journal.pone.0157063 (PMC4898697; doi:10.1371/journal.pone.0157063)
Supplement: S1 Table — (DOCX) [file pone.0157063.s003.docx]

**S1 Table.**

| **Time point** | **Status** | **High FCM** | **Low FCM** | **Oral iron** | **Total** |
| --- | --- | --- | --- | --- | --- |
| **Baseline** | Evaluable | 17 | 16 | 28 | 61 |
| Week 4 | No hepcidin value with time window |  |  | 2 |  |
|  | Evaluable | 17 | 16 | 26 | 59 |
| Week 8 | Other anemia management | 0 | 0 | 1 | 1 |
|  | No hepcidin value with time window | 1 | 0 | 3 | 4 |
|  | Evaluable | 16 | 16 | 24 | 56 |
| Week 12 | Other anemia management | 0 | 0 | 1 | 1 |
|  | No hepcidin value with time window | 1 | 0 | 2 | 3 |
|  | Evaluable | 16 | 16 | 25 | 57 |
| Week 24 | Other anemia management | 2 | 1 | 4 | 7 |
|  | No hepcidin value with time window | 0 | 1 | 5 | 6 |
|  | Evaluable | 15 | 14 | 19 | 48 |
| Week 36 | Other anemia management | 2 | 2^a^ | 6 | 10 |
|  | No hepcidin value with time window | 2 | 2 | 6 | 10 |
|  | Evaluable | 13 | 12 | 16 | 41 |
| Week 52 | Other anemia management | 2 | 2^a^ | 6 | 9 |
|  | No hepcidin value with time window | 5 | 6 | 12 | 23 |
|  | Evaluable | 10 | 8 | 10 | 28 |

^a^ 1 patient also discontinued the study

FCM, ferric carboxymaltose
